# Nonlinear associations of nighttime sleep and protective napping with depressive symptoms: a cross-sectional analysis from the China Family Panel Studies

**DOI:** 10.3389/fpsyt.2025.1680714

**Published:** 2025-10-24

**Authors:** Yanliqing Song, Yue Liu, Li Liu

**Affiliations:** ^1^ College of Sports, Nanjing Tech University, Nanjing, China; ^2^ School of Athletic Performance, Shanghai University of Sport, Shanghai, China

**Keywords:** COVID-19 pandemic, depression, nap time, nighttime sleep, health promotion

## Abstract

**Objective:**

This study aims to explore the relationship between nap duration, nighttime sleep, and depression among Chinese residents and determine recommended sleep durations to provide scientific evidence for the prevention and control of depression.

**Methods:**

Based on the 2020 China Family Panel Studies, demographic data, health, and lifestyle information was obtained from the study subjects. A total of 6,795 valid samples were included. Logistic regression, restricted cubic splines, and stratified linear regression analyses were used to examine the associations between sleep behaviors and depression, including subgroup analyses by health status and age categories.

**Results:**

A U-shaped dose–response relationship was observed between nighttime sleep and depressive symptoms (P-nonlinear < 0.001), with the lowest likelihood of depression occurring around 8.5 hours of sleep. A nap duration of 30–90 minutes was associated with a lower likelihood of depression, with no evidence of a nonlinear relationship (P-nonlinear = 0.889). Subgroup analyses revealed that nighttime sleep of 7–9 hours was protective against depression among individuals with self-rated general health or chronic diseases. Age-stratified analyses showed that sleep behaviors had stronger protective effects in young adults (<30 years), whereas depression in middle-aged and older adults (≥30 years) was more influenced by chronic disease status and education level.

**Conclusion:**

Nighttime sleep of 7–9 hours and nap duration of 30–90 minutes are associated with reduced depressive symptoms; however, their effects vary across health and age subgroups. These findings highlight the importance of tailoring sleep recommendations to individual characteristics for effective mental health promotion.

## 1 Introduction

Depression is a widespread mental health disorder, affecting an estimated 280 million people globally (1). As one of the leading causes of disability, the prevention and intervention strategies for depression are a pressing focus in global public health. Notably, the prevalence of depression is not limited to middle-aged and elderly populations; its incidence among young adults is also significant (2). For instance, a survey conducted in the United States revealed that the prevalence of depressive symptoms among college students is as high as 85%, highlighting the severity and urgency of this issue, which requires further attention and research (3).

The global burden of depression has significantly increased during the COVID-19 pandemic. According to the latest data, the number of severe depression cases worldwide has risen by 52.3 million, representing an increase of 27.6% (4). Across the 204 countries and regions involved, the incidence of severe depression has risen to 3,152.9 cases per 100,000 people (5). Stress factors related to the pandemic, including unemployment, the death of loved ones, social isolation, and the compound effects of multiple stressors, have had a severe negative effect on global mental health (6). In the United States, the prevalence of depressive symptoms during COVID-19 tripled compared to pre-pandemic levels (7).

With hundreds of thousands of new COVID-19 cases being reported daily, the pandemic has profound affected human diet, daily outdoor activities, and overall lifestyle habits (8). Public health measures implemented to control the pandemic, such as advising individuals to avoid crowded public places (e.g., playgrounds, basketball courts, and football fields), inevitably led to reduced opportunities for physical exercise. Existing research has clearly indicated a close link between the frequency of physical exercise and the incidence of anxiety and depressive symptoms (9).

As the number of new COVID-19 cases continues to rise, necessary control measures such as lockdowns and restrictions on population movement have been implemented to curb the spread of the pandemic. However, these measures may exacerbate mental stress due to the restriction of personal freedom (10). Studies suggest that an individual’s geographical proximity to disaster events and the degree of personal effect experienced during natural disasters or epidemics may affect post-event mental health recovery. Furthermore, the influence of economic and social changes on mental health may be delayed (11).

Although direct exposure to the COVID-19 virus does not appear to have a significant direct effect on mental health symptoms, the long-term increase in COVID-19-related occupational and social barriers may lead to significant changes in mental health, particularly due to social disruption, work-related obstacles, and economic difficulties (12). Therefore, from a public health perspective, understanding and identifying modifiable risk factors for depression is crucial for developing effective prevention and intervention strategies.

Depression is recognized as a complex mental disorder, generally resulting from the combined interaction of genetic and environmental factors (13). Moreover, lifestyle is a key determinant of its occurrence (14). As a vital element of health, sleep has recently garnered increased attention in academic research. Studies have indicated that sleep duration correlates significantly with overall mortality, cardiovascular diseases, and the prevalence of chronic conditions such as metabolic syndrome (15, 16). Insufficient sleep duration and poor quality are considered major contributors to mental health disorders. Consequently, the relationship between sleep, as a modifiable lifestyle factor, and depression has been examined in numerous studies (17, 18).

Researchers have extensively explored the role of sleep duration in the development of depression. A prospective study found that short and long sleep durations are significantly associated with an increased risk of depression in adults (19). This perspective is corroborated by subsequent research, which suggests that insufficient or excessive sleep may increase the incidence of depression (20). However, the data collection for these studies was completed before the outbreak of the COVID-19 pandemic.

Therefore, this study utilizes the latest China Family Panel Studies (CFPS) data, which were obtained during the COVID-19 pandemic. This dataset was selected because it best represents the characteristics of depression among Chinese residents at this stage. This study aims to explore the relationship between nap duration, nighttime sleep, and depression among residents and determine recommended sleep durations. This will provide scientific evidence for the prevention and treatment of depression.

## 2 Methods

### 2.1 Study population

The CFPS is a nationwide, comprehensive project that collects individual, family, and community data via biennial follow-up surveys to track changes in China’s socioeconomic, demographic, educational, and health fields. Data for this study were derived from the 2020 CFPS project. The project employed computer-assisted survey techniques to meet diverse design needs, improve survey efficiency, and ensure data quality. Samples with missing demographic variables or missing Center for Epidemiologic Studies Depression Scale (CESD) scores were excluded. Ultimately, 6,795 samples were included in this study.

### 2.2 Outcome

Nighttime sleep duration was assessed using the question: “Excluding nap duration, how many hours do you usually sleep each day on average, representing a typical workday and rest day?” Based on established benefits of 7–9 hours of sleep (21), participants were categorized into three types groups: short nighttime sleep (<7 hours), moderate nighttime sleep (7–9 hours; reference group), and long nighttime sleep (>9 hours).

Nap duration was assessed using the question: “How many minutes do you usually nap?” Participants were categorized into four groups: no nap (0 minute), short nap (<30 minutes), moderate nap (30–90 minutes), and long nap (>90 minutes). These cut-points were selected by referencing epidemiological literature on daytime napping (22).

### 2.3 Explanatory variable

Depression was measured using the CESD. This scale, developed by Radloff at the National Institute of Mental Health, has been widely used to assess depression levels (23). The CFPS 2020 used the eight-item version of the CESD, which includes items such as: “I felt depressed,” “I felt happy,” and “I felt that life was not worth living.” Responses were scored as 0 = rarely or never, 1 = not often, 2 = sometimes or half the time, and 3 = most of the time, with reverse-scored items transposed. The summed scores of the eight items ranged from 5 to 24. Higher total scores indicate a greater risk of depression, with scores of ≥9 indicating clinically significant depressive symptoms (24). The CESD-8 is recognized as an effective and reliable tool for assessing depressive symptoms in adults (25). The reliability coefficient for the CESD-8 scale in this study was examined, yielding a Cronbach’s alpha greater than 0.7, which indicates good internal consistency.

### 2.4 Other covariates

The analysis incorporated a set of potential confounding variables.

Sociodemographic data included age, gender (female or male), education level (illiterate, primary school, middle school, or high school and above), region (urban or rural), and marital status (married or single).

Lifestyle factors included smoking (yes or no), drinking (yes or no), and frequency of physical activity (0 times a week, 1–2 times a week, 3–4 times a week, 5–6 times a week, or every day).

Health status included health self-assessment (bad, fair, or good) and chronic disease (no or yes).

### 2.5 Statistical methods

Statistical analyses were performed using R 4.2 software. Qualitative data were summarized using frequencies and percentages (%), with group comparisons conducted using the χ2 test or Fisher’s exact test. Quantitative data conforming to a normal distribution were expressed as mean ± standard deviation (x ± s), and group comparisons utilized the t-test. Logistic regression models were constructed, employing nap duration and nighttime sleep as the reference groups. The variance inflation factor was used to assess multicollinearity among independent variables, and the Box–Tidwell tested the linear relationship between continuous independent variables and the logit of depression. Three models were constructed. Model 1 included only nighttime and nap duration, Model 2 controlled for demographic variables, and Model 3 included all covariates. Restricted cubic spline plots were generated to explore the dose–response relationship between participants’ sleep duration and depression. Stratified logistic regression analysis and interaction effect analysis determined whether the association between sleep duration and depression was dependent on baseline or demographic factors. P-values were used for two-sided tests, with P < 0.05 considered statistically significant.

Additional stratified linear regression analyses were performed to further examine the relationship between sleep duration and depressive symptoms across nap and nighttime sleep subgroups, as well as age categories. In these models, which used CESD scores as the continuous dependent variables, each was adjusted for age, gender, education level, and chronic disease status. A total of 10 stratified models were constructed, with subgroup-specific regression coefficients, confidence intervals, and P-values reported. These models were designed to isolate the independent effects of sleep behaviors while controlling for key demographic and health-related covariates.

The primary analysis initially treated depression status as a binary variable, utilizing logistic regression models. However, when stratified analyses were performed by nap and nighttime sleep groups, several models encountered convergence failures or perfect separation. This was attributable to limited sample sizes or imbalanced group distributions within those specific strata. To address these methodological issues while preserving subgroup distinctions, the CESD total scores were subsequently used as continuous dependent variables, and linear regression models were employed. This change enhanced model stability and enabled a more nuanced interpretation of the relationship between sleep behaviors and depression severity.

For the age-stratified analyses, participants were divided into three groups based on the actual sample age distribution and developmental relevance: young adults (<30 years), middle-aged adults (30–44 years), and older adults (≥45 years). This classification ensured sufficient sample size and developmental relevance across subgroups.

## 3 Results

### 3.1 Participant characteristics

The valid sample consisted of 6795 participants, of which 3411 were female (50.20%). The average age was 31.80 ± 8.67 years, and 1353 participants (19.9%) exhibited depressive symptoms. Differences in nap sleep time, nighttime sleep, region, level of education, health self-assessment, chronic disease, and frequency of physical activity were statistically significant (P<0.05). Specific basic characteristics are shown in **Table 1**.

**Table 1:** Basic characteristics of study participants (n=6795).

Variables	Total (n = 6795)	Normal (n = 5442)	Depression (n = 1353)	Statistic	*P*
Age, Mean ± SD	31.80 ± 8.67	31.62 ± 8.79	32.54 ± 8.17	t=-3.67	<.001
Nap Duration, M (Q_1_, Q_3_)	20.00 (0.00, 60.00)	30.00 (0.00, 60.00)	0.00 (0.00, 60.00)	Z=-2.35	0.019
Nighttime sleep, Mean ± SD	7.59 ± 1.22	7.64 ± 1.17	7.36 ± 1.38	t=6.97	<.001
Depression, Mean ± SD	5.33 ± 3.94	3.93 ± 2.89	10.94 ± 2.24	t=-96.70	<.001
Gender, n(%)				χ²=0.70	0.401
female	3411 (50.20)	2718 (49.94)	693 (51.22)		
male	3384 (49.80)	2724 (50.06)	660 (48.78)		
Region, n(%)				χ²=0.67	0.414
countryside	5425 (79.84)	4334 (79.64)	1091 (80.64)		
city	1370 (20.16)	1108 (20.36)	262 (19.36)		
Level of education n(%)				χ²=49.53	<.001
illiterate	423 (6.23)	300 (5.51)	123 (9.09)		
elementary school	888 (13.07)	666 (12.24)	222 (16.41)		
Junior high school	2893 (42.58)	2329 (42.80)	564 (41.69)		
high school and above	2591 (38.13)	2147 (39.45)	444 (32.82)		
Marital status, n(%)				χ²=0.05	0.819
single	2001 (29.45)	1606 (29.51)	395 (29.19)		
married	4794 (70.55)	3836 (70.49)	958 (70.81)		
Health self-assessment, n(%)				χ²=253.63	<.001
bad	2834 (41.71)	2477 (45.52)	357 (26.39)		
fair	3097 (45.58)	2417 (44.41)	680 (50.26)		
good	864 (12.72)	548 (10.07)	316 (23.36)		
Chronic disease, n(%)				χ²=39.87	<.001
No	6405 (94.26)	5178 (95.15)	1227 (90.69)		
yes	390 (5.74)	264 (4.85)	126 (9.31)		
Frequency of physical activity, n(%)				χ²=16.97	0.002
0 times a week	5058 (74.44)	3995 (73.41)	1063 (78.57)		
1~2 times a week	689 (10.14)	571 (10.49)	118 (8.72)		
3~4 times a week	376 (5.53)	313 (5.75)	63 (4.66)		
5~6 times a week	52 (0.77)	40 (0.74)	12 (0.89)		
every day	620 (9.12)	523 (9.61)	97 (7.17)		
Smoking, n(%)				χ²=12.39	<.001
no	4911 (72.27)	3985 (73.23)	926 (68.44)		
yes	1884 (27.73)	1457 (26.77)	427 (31.56)		
Drink, n(%)				χ²=3.40	0.065
no	6061 (89.20)	4873 (89.54)	1188 (87.80)		
yes	734 (10.80)	569 (10.46)	165 (12.20)		
Nighttime sleep, n(%)				χ²=70.59	<.001
< 7h	1093 (16.09)	149 (2.74)	85 (6.28)		
7-9 h	4687 (68.98)	2187 (40.19)	632 (46.71)		
>9 h	1015 (14.94)	3106 (57.07)	636 (47.01)		
Nap sleep time, n(%)				χ²=5.95	0.114
0 min	3230 (47.53)	2550 (46.86)	680 (50.26)		
<30 min	1033 (15.20)	828 (15.21)	205 (15.15)		
30-90 min	1981 (29.15)	1617 (29.71)	364 (26.90)		
>90 min	551 (8.11)	447 (8.21)	104 (7.69)		

t, t-test; Z, Mann-Whitney test; χ², Chi-square test; M, Median; Q_1_, 1st Quartile; Q_3_, 3st Quartile; SD, standard deviat.

### 3.2 Logistic regression of the relationship between sleep duration and depression

For the diagnosis of multicollinearity, we eliminated variables with VIF values greater than 10 to avoid multicollinearity. The Box-Tidwell test results showed that the continuous independent variables (nighttime sleep, nap sleep time) had a linear relationship with the dependent variable (depression) (P >0.05). The results of the logistic regression model analysis showed that, after controlling for all covariates in Model 3, participants with nighttime sleep ≥7 hours were found to have a protective factor against the likelihood of depression compared to those with nighttime sleep <7 hours. Compared to participants without a napping habit, those with nap times of 30–90 minutes were found to have a protective factor against the likelihood of depression (**Table 2**).

**Table 2:** Logistic regression table of the relationship between sleep duration and depression.

Variables	P	OR (95%CI)	P	OR (95%CI)	P	OR (95%CI)
Nighttime sleep (h)						
< 7h		1.00 (Reference)		1.00 (Reference)		1.00 (Reference)
7–9 h	**<.001**	0.64 (0.52 ~ 0.80)	**<.001**	0.63 (0.51 ~ 0.78)	**<.001**	0.68 (0.55 ~ 0.85)
>9 h	<.001	0.60 (0.47 ~ 0.78)	<.001	0.57 (0.44 ~ 0.74)	**<.001**	0.63 (0.49 ~ 0.83)
Nap time (min)						
0 min		1.00 (Reference)		1.00 (Reference)		1.00 (Reference)
<30 min	0.788	1.03 (0.83 ~ 1.27)	0.710	1.04 (0.84 ~ 1.28)	0.854	1.02 (0.83 ~ 1.26)
30–90 min	**0.021**	0.83 (0.71 ~ 0.97)	**0.028**	0.84 (0.71 ~ 0.98)	**0.032**	0.84 (0.71 ~ 0.98)
>90 min	0.062	0.79 (0.61 ~ 1.01)	0.055	0.78 (0.61 ~ 1.01)	0.076	0.80 (0.62 ~ 1.02)

Model 1 includes nighttime and nap time of the elderly, model 2 controls for demographic variables, and model 3 has all covariates. OR, Odds Ratio; CI, Confidence Interval.

Values in bold represent statistically significant associations, defined as P-values less than 0.05.

### 3.3 Dose–response relationship between nighttime sleep and depression

**Figure 1:**
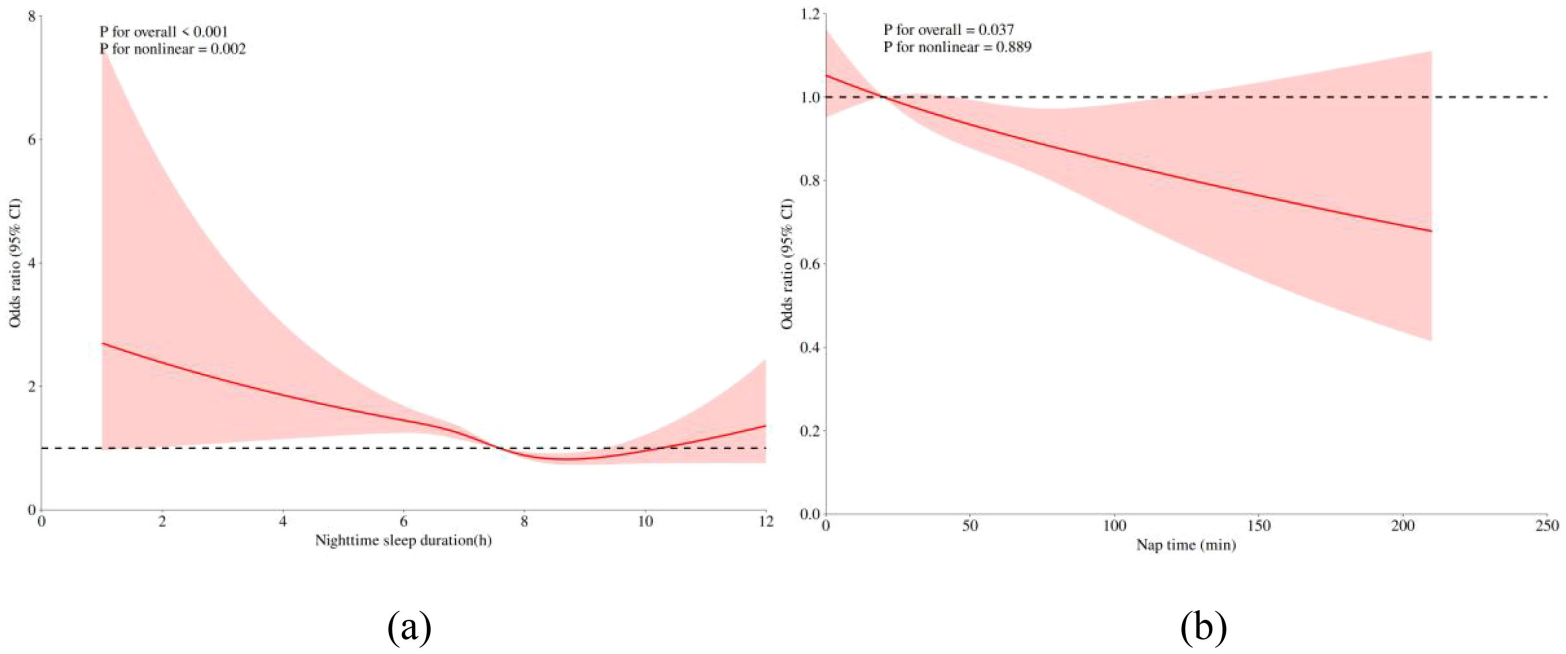
**(A)** Dose-response relationship between nighttime sleep and depression in older adults, **(B)** Dose-response relationship between nap time and depression in older adults. The x-axis represents sleep duration. The y-axis represents the OR values calculated by the model. The shaded area represents the 95% confidence interval (Overall trend test: P < 0.001; Nonlinear trend test: P < 0.001).

The dose-response relationship analysis between nighttime sleep and depression indicated a U-shaped relationship (P-nonlinear <0.001), with the likelihood of depression decreasing from 7.5 hours of sleep to about 8.5 hours (**Figure 1**). The lowest likelihood of depression was observed when participants had around 8.5 hours of sleep. The dose-response relationship analysis between nap sleep time and depression indicated no nonlinear relationship (P-nonlinear = 0.889) (**Figure 1**), also supported by the results in **Table 2**.

### 3.4 Subgroup analysis of the effect of sleep duration on depression

Stratified logistic regression analysis and interaction effect analysis were used to determine whether the association between sleep duration and depression depended on baseline or demographic factors. The association between nighttime sleep of 7–9 hours and depression demonstrated a significant interaction with health self-assessment and chronic disease (interaction P-values <0.05). This finding indicates that, for individuals with a general health self-assessment or chronic diseases, a 7–9 hour sleep duration may have a protective effect against the likelihood of depression. Nighttime sleep of 7–9 hours was thus only associated with the likelihood of depression in certain subgroups. These subgroup-specific associations are further supported by the logistic regression results shown in **Table 3**.

**Table 3:** Subgroup analysis of the impact of nighttime sleep on depression.

Variables	n (%)	Normal	Depression	OR (95%CI)	P	P for interaction
All patients	6795 (100.00)	1839/2108	3995/4687	0.84 (0.73 ~ 0.98)	0.028	
Region						0.351
countryside	5425 (79.84)	1476/1677	3245/3748	0.88 (0.74 ~ 1.05)	0.146	
city	1370 (20.16)	363/431	750/939	0.74 (0.55 ~ 1.01)	0.056	
Level of education						0.585
illiterate	423 (6.23)	148/167	233/256	1.30 (0.68 ~ 2.47)	0.422	
elementary school	888 (13.07)	279/317	497/571	0.91 (0.60 ~ 1.39)	0.676	
Junior high school	2893 (42.58)	784/894	1716/1999	0.85 (0.67 ~ 1.08)	0.179	
high school and above	2591 (38.13)	628/730	1549/1861	0.81 (0.63 ~ 1.03)	0.081	
Health self-assessment						**0.015**
bad	2834 (41.71)	693/854	1611/1980	1.01 (0.83 ~ 1.25)	0.892	
fair	3097 (45.58)	838/923	1882/2174	0.65 (0.51 ~ 0.84)	**0.001**	
good	864 (12.72)	308/331	502/533	1.21 (0.69 ~ 2.11)	0.504	
Chronic disease						**0.040**
No	6405 (94.26)	1698/1962	3776/4443	0.88 (0.75 ~ 1.03)	0.103	
yes	390 (5.74)	141/146	219/244	0.31 (0.12 ~ 0.83)	**0.020**	
Frequency of physical activity						0.628
0 times a week	5058 (74.44)	1415/1605	2970/3453	0.83 (0.69 ~ 0.99)	0.036	
1~2 times a week	689 (10.14)	172/201	401/488	0.78 (0.49 ~ 1.23)	0.279	
3~4 times a week	376 (5.53)	88/99	235/277	0.70 (0.34 ~ 1.42)	0.322	
5~6 times a week	52 (0.77)	11/14	30/38	1.02 (0.23 ~ 4.57)	0.977	
every day	620 (9.12)	153/189	359/431	1.17 (0.75 ~ 1.83)	0.479	

OR, Odds Ratio; CI, Confidence Interval.

Values in bold represent statistically significant associations, defined as P-values less than 0.05.

Conversely, the association between a nap duration of 30–90 minutes and depression showed no significant interaction with region, education level, health self-assessment, chronic disease status, or frequency of physical activity (interaction P-values >0.05). This suggests that the effect of nap duration on depression did not differ across these baseline or demographic factors; the association between appropriate nap duration and depression risk was consistent across all stratified subgroups, as shown in **Table 4**.

**Table 4:** Subgroup analysis of the impact of nap time on depression.

Variables	n (%)	Normal	Depression	OR (95%CI)	P	P for interaction
All patients	6795 (100.00)	4162/4814	1672/1981	0.85 (0.73 ~ 0.98)	**0.027**	
Region						0.810
countryside	5425 (79.84)	3362/3840	1359/1585	0.85 (0.72 ~ 1.01)	0.071	
city	1370 (20.16)	800/974	313/396	0.82 (0.61 ~ 1.10)	0.184	
Level of education						0.271
illiterate	423 (6.23)	296/328	85/95	0.92 (0.43 ~ 1.95)	0.825	
elementary school	888 (13.07)	577/647	199/241	0.57 (0.38 ~ 0.87)	**0.009**	
Junior high school	2893 (42.58)	1761/2029	739/864	0.90 (0.72 ~ 1.13)	0.366	
high school and above	2591 (38.13)	1528/1810	649/781	0.91 (0.72 ~ 1.14)	0.400	
Health self-assessment						0.384
bad	2834 (41.71)	1649/2006	655/828	0.82 (0.67 ~ 1.00)	0.055	
fair	3097 (45.58)	1920/2181	800/916	0.94 (0.74 ~ 1.18)	0.588	
good	864 (12.72)	593/627	217/237	0.62 (0.35 ~ 1.10)	0.105	
Chronic disease						0.623
No	6405 (94.26)	3935/4568	1539/1837	0.83 (0.72 ~ 0.96)	**0.015**	
yes	390 (5.74)	227/246	133/144	1.01 (0.47 ~ 2.19)	0.976	
Frequency of physical activity						0.381
0 times a week	5058 (74.44)	3218/3685	1167/1373	0.82 (0.69 ~ 0.98)	**0.030**	
1~2 times a week	689 (10.14)	396/471	177/218	0.82 (0.54 ~ 1.24)	0.347	
3~4 times a week	376 (5.53)	208/239	115/137	0.78 (0.43 ~ 1.41)	0.408	
5~6 times a week	52 (0.77)	26/32	15/20	0.69 (0.18 ~ 2.66)	0.592	
every day	620 (9.12)	314/387	198/233	1.32 (0.85 ~ 2.04)	0.223	

OR, Odds Ratio; CI, Confidence Interval.

Values in bold represent statistically significant associations, defined as P-values less than 0.05.

To gain a deeper understanding of the relationship between sleep duration and depressive symptoms, 10 stratified linear regression models were constructed. These models spanned various nap and nighttime sleep subgroups, in addition to age categories. CESD scores were utilized as the dependent variable in all models, which were adjusted for age, gender, education level, and chronic disease status.

Significant associations emerged in multiple subgroups: Moderate nap duration was consistently associated with lower CESD scores in the short nighttime sleep group (β = −0.676, 95% CI: −1.028 to −0.325, p < 0.001) and among young adults (β = −0.457, 95% CI: −0.700 to −0.215, p < 0.001). Nighttime sleep duration showed protective effects in the no-nap group, with normal (β = −1.029, 95% CI: −1.325 to −0.733, p < 0.001) and long sleep durations (β = −0.912, 95% CI: −1.499 to −0.326, p = 0.002) linked to lower depression scores.

Education level was identified as a robust protective factor across multiple subgroups, including short nap (β = −0.395, p < 0.001), short nighttime sleep (β = −0.365, p < 0.001), middle-aged adults (β = −0.400, p = 0.010), and older adults (β = −1.723, p = 0.042). Conversely, chronic disease was positively associated with depressive symptoms in nearly all subgroups, with the strongest effect observed in the short nighttime sleep group (β = 1.907, 95% CI: 1.306 to 2.507, p < 0.001). The full results of the stratified linear regression models are presented in **Table 5**.

**Table 5:** Statistically significant results from stratified linear regression models on CESD scores.

Analysis subgroup	Variable	Estimate	95% CI	P-value
Short sleep group	Moderate nap	−0.676	[−1.028, −0.325]	**<0.001**
Short sleep group	Education	−0.365	[−0.551, −0.179]	**<0.001**
Short sleep group	Chronic disease	1.907	[1.306, 2.507]	**<0.001**
No nap group	Normal sleep	−1.029	[−1.325, −0.733]	**<0.001**
No nap group	Long sleep	−0.912	[−1.499, −0.326]	**0.002**
Short nap group	Normal sleep	−0.652	[−1.125, −0.179]	**0.007**
Moderate nap group	Normal sleep	−0.595	[−0.947, −0.242]	**<0.001**
Young adults	Moderate nap	−0.457	[−0.700, −0.215]	**<0.001**
Young adults	Normal sleep	−0.987	[−1.202, −0.772]	**<0.001**
Young adults	Chronic disease	1.915	[1.425, 2.405]	**<0.001**
Middle-aged adults	Education	−0.400	[−0.703, −0.098]	**0.010**
Middle-aged adults	Chronic disease	0.974	[0.034, 1.914]	**0.042**
Older adults	Education	−1.723	[−3.379, −0.067]	**0.042**

All models were adjusted for age, gender, education, and chronic disease.

Values in bold represent statistically significant associations, defined as P-values less than 0.05.

## 4 Discussion

This study revealed a U-shaped dose–response relationship between nighttime sleep and depressive symptoms. Specifically, the probability of depressive symptoms significantly decreased when nighttime sleep increased from 7.5 hours to 8.5 hours. This result aligns with a cross-sectional study involving 1,788 American adults (aged 19–89 years), which also showed a U-shaped association between sleep duration and depressive symptoms (26). Compared to those with nighttime sleep of less than 6 hours, individuals with sufficient nighttime sleep (not less than 6 hours) had a lower risk of depression. Furthermore, research conducted in southwestern China, involving 44,900 Han adults aged 30–79 years, similarly found that participants with nighttime sleep less than 7 hours had a higher odds ratio for depression (OR: 1.47, 95% CI: 1.31–1.65) compared to those with 7–8 hours of sleep. However, this Chinese study reported no significant association between nighttime sleep exceeding 9 hours and depressive symptoms in either the overall population or subgroup analyses (27)—a finding supported by other literature that suggests no clear link between long sleep duration and depression (28). Conversely, a meta-analysis of seven prospective studies indicated a significant association between short sleep duration and depression risk, with a risk ratio of 1.31 compared to normal sleep duration (29). Some cross-sectional studies have also identified associations between short (≤6 hours) and long sleep durations (≥9 hours) and an increased risk of depressive symptoms (30).

The observed inconsistency regarding the relationship between nighttime sleep and depressive symptoms across various studies may be attributed to several factors. (1) Differences in the methods used to adjust for potential confounding factors may introduce biased results. (2) Moreover, varied definition of sleep duration across studies may compromise the comparability of findings. (3) Differences in the age distribution of study subjects may also influence the observed relationship between sleep duration and depressive symptoms. (4) Furthermore, the relatively low proportion of participants with long sleep duration (14.94%) may result in insufficient statistical power to accurately assess the relationship between long sleep duration and depressive symptoms. (5) In addition, a potential bidirectional relationship between depression and sleep may cause differences between results from prospective cohort studies and cross-sectional studies. (6) Finally, urban–rural differences and socioeconomic status (SES) disparities may significantly affect the pathogenesis of depression. Given that sleep and depression are associated with specific health behaviors and SES, these factors may confound the sleep–depression association [e.g., long sleep duration is associated with lower SES, reduced physical activity, and obesity, all of which are risk factors for depression (31)]. Therefore, these potential confounding variables must be carefully considered when interpreting the relationship between sleep duration and depressive symptoms.

This study also found that participants with a nap duration of 30–90 minutes exhibited a lower likelihood of depression, which supports the potential role of napping in mood regulation. Previous studies have similarly indicated that short daytime naps are not significantly associated with depressive symptoms; however, excessively long nap duration have been identified as an independent risk factor for depression (32). Specifically, compared to moderate nappers, long nappers had a significantly higher incidence of depressive symptoms (OR = 1.32, 95% CI: 1.19–1.45) (33). This finding underscores the importance of nap duration in mental health and suggests that an appropriate nap duration may have a protective effect on emotional health.

Several possible explanatory mechanisms link sleep status and depressive symptoms. First, insufficient sleep may lead to daytime fatigue, subsequently causing excessive daytime sleepiness and the disruption of circadian rhythms, all of which may increase the risk of depressive symptoms. Individuals with shorter sleep durations may experience insufficient rest and increased perceived stress—an established risk factor for depressive symptoms (34). Second, inflammation is a key correlate of depression. Studies have shown that short sleep duration and insomnia symptoms are associated with elevated levels of inflammatory cytokines, such as C-reactive protein and interleukin-6 (35). Increasing evidence indicates that short and long sleep durations are associated with elevated levels of inflammation, a phenomenon particularly evident in individuals with depression (36). Chronic low-grade inflammation may therefore serve as an important biological pathway linking sleep and depressive symptoms. Notably, inflammation is not only associated with depression and stress disorders but also plays a crucial role in their pathophysiology (37). Increased inflammation may further promote the activation of the kynurenine pathway, resulting in reduced serotonin levels, which is closely related to the pathophysiological mechanisms of depression (38).

Additionally, sleep quality and duration may influence the composition of the gut microbiota (39). The microbiota–gut–brain axis is an interactive system implicated in various neurological diseases (40). Studies have shown that sleep disorders may alter the levels of brain-derived neurotrophic factor, which plays a key role in the pathophysiology of stress-related mood disorders (41). The hypothalamic–pituitary–adrenal (HPA) axis serves as an important pathway for understanding the neurobiology of stress responses (42). Activation of the HPA axis occurs when corticotropin-releasing hormone, released by the hypothalamus, stimulates the anterior pituitary to secrete adrenocorticotropic hormone, which, in turn, promotes the adrenal cortex to release cortisol into the bloodstream (43). Conversely, a decline in sleep quality may enhance the stress reactivity of the HPA axis.

Furthermore, adequate sleep helps in elevating melatonin levels, a molecule with multifaceted regulatory functions that can help alleviate depressive symptoms. Finally, research connecting sleep and the immune system suggests that sleep can optimize immune defense, and immune cell signaling may induce sleep. Immune activation and cytokines may play a role in the occurrence of depressive symptoms in certain individuals. These findings collectively underscore the important role of sleep in maintaining mental health and preventing depressive symptoms.

The study conducted a subgroup analysis of the sleep–depression relationship between to explore whether this association is influenced by baseline or demographic factors. Stratified logistic regression analysis and interaction effect analysis revealed a significant interaction between nighttime sleep of 7–9 hours and depression, particularly related to health self-assessment and chronic disease status. Specifically, for individuals with general health self-assessment or chronic diseases, maintaining 7–9 hours of nighttime sleep may have a protective effect, reducing the risk of depression. This result aligns with previous quantile regression studies, including 55,954 adults from two national surveys, which found that the association between short sleep duration and depression was stronger in individuals with chronic diseases (44). Thus, the association between short sleep duration and depression appears to be modified by chronic diseases. Sleep problems, chronic diseases, and depression are interrelated, with each increasing the risk of occurrence and worsening of the others. A review further indicated that alterations in mesolimbic dopaminergic function are neurobiological factors associated with this triad (45). However, the exact nature of these associations remains to be fully elucidated. Further research is necessary to clarify the underlying physiological mechanisms connecting sleep, depression, and chronic diseases. This finding suggests that the role of sleep duration in preventing depression may vary depending on an individual’s health status, emphasizing the importance of considering individual differences in clinical practice. The significant association between 7–9 hours of nighttime sleep and the likelihood of depression was observed only in certain subgroups, indicating that the effect of sleep on mental health is not universally applicable but is influenced by specific demographic characteristics. Therefore, future research and intervention strategies must consider the particularities of these subgroups to prevent and treat depression more effectively.

In addition to subgroup analyses by health status and nap duration, this study also performed stratified linear regression analyses across three age groups: young adults (<30 years), middle-aged adults (30–44 years), and older adults (≥45 years). The results demonstrated that the association between sleep duration and depressive symptoms varied significantly across age groups. Specifically, moderate nap duration (30–90 minutes) and normal nighttime sleep (7–9 hours) were strongly linked to lower CESD scores in young adults, whereas chronic disease status and education level showed a stronger association with depression in middle-aged and older adults.

These findings are consistent with prior research. A narrative review concluded that short and long sleep durations are risk factors for depression in older adults, often co-occurring with chronic diseases and cognitive decline (46). These age-related vulnerabilities may explain why sleep optimization shows stronger protective effects in younger populations, whereas the depressive symptoms of older adults are more influenced by comorbid conditions.

Age-related physiological changes, lifestyle patterns, and stress exposures likely contribute to these differences. For example, younger individuals often experience greater circadian instability, academic and occupational stress, and social irregularities, making them more sensitive to sleep deprivation and mood fluctuations. By contrast, older adults may suffer from fragmented sleep, reduced sleep efficiency, and a higher prevalence of chronic conditions, such as cardiovascular diseases, diabetes, and neurodegeneration, which can exacerbate depressive symptoms independently of sleep duration.

Therefore, sleep interventions should be tailored to developmental stages. Younger individuals may benefit more directly from behavioral sleep optimization, such as consistent sleep schedules and sleep hygiene education. By contrast, older adults likely require integrated approaches addressing sleep, chronic disease, and cognitive health simultaneously. These findings underscore the importance of age-specific strategies in clinical practice and public health planning. They also suggest that future research should further explore biological mechanisms—such as inflammation, HPA axis dysregulation, and neuroendocrine changes—that may mediate the sleep–depression relationship across the lifespan.

Conversely, this study demonstrated that the association between nap duration of 30–90 minutes and the risk of depression showed no interaction across subgroups defined by region, education level, health self-assessment, chronic disease status, and frequency of physical activity. This suggests that the beneficial effect of appropriate nap duration on reducing the risk of depression is consistent across different demographic characteristics and is not influenced by these factors. This finding supports the notion of napping as a universally beneficial health behavior and provides a theoretical basis for future sleep interventions. Overall, these findings offer new directions for future research, suggesting that sleep interventions must consider individual differences in health status and sleep habits. In addition, public health strategies should promote appropriate sleep habits to reduce the incidence of depression.

Despite providing valuable insights, this study has several limitations. First, due to the cross-sectional nature of the study design, the causal relationship between sleep duration and depressive symptoms cannot be determined; longitudinal designs are necessary to explore the temporal relationship. Second, the study’s reliance on self-reported sleep data introduces the risk of subjective bias. Future research should consider incorporating objective sleep monitoring techniques, such as polysomnography or wearable devices, to improve data accuracy. In addition, this study did not account for individual differences, such as genetic factors, life events, and personality traits, all of which may influence the sleep–depression relationship. Future research should therefore explore these potential mediating or moderating variables.

## 5 Conclusion

This study established a complex and nonlinear relationship between sleep duration and depressive symptoms among Chinese residents. A U-shaped dose–response pattern was observed for nighttime sleep, where a duration of 7–9 hours was associated with the lowest likelihood of depression. Furthermore, a nap duration of 30–90 minutes consistently exhibited a protective effect across all analyzed demographic and health subgroups. Importantly, stratified analyses revealed that the mental health benefits of sleep behaviors are age-dependent: younger adults experienced stronger protective effects from optimal sleep, whereas the presence of chronic disease and education level were more influential determinants of depression in middle-aged and older adults. These findings underscore the critical need for age-sensitive and health-specific sleep recommendations in mental health promotion. Public health strategies must prioritize nighttime sleep and nap hygiene, specifically tailoring interventions to individual characteristics to prevent depression more effectively.

## Data Availability

Publicly available datasets were analyzed in this study. This data can be found here: https://www.isss.pku.edu.cn/cfps/sjzx/gksj/index.htm.
